# Perspectives Toward Person-Centered Long-Term Care in China

**DOI:** 10.1093/geroni/igab046.612

**Published:** 2021-12-17

**Authors:** Jing Wang, Kirsten Corazzini, Bei Wu

**Affiliations:** 1 Fudan University, Chapel Hill, North Carolina, United States; 2 University of Maryland School of Nursing, Baltimore, Maryland, United States; 3 New York University, New York, New York, United States

## Abstract

Health care aides provide direct care for older residents with advanced dementia in long-term care facilities. This study aims to understand care aides’ perceptions of what is ‘good’ care, what is person-centered care, and how to provide person-centered care for older residents with advanced dementia, as preparatory work of the WE-THRIVE consortium’s efforts to develop internationally-relevant common data elements of person-centered dementia care and launch comparative research in LMICs. Semi-structured interviews were conducted with health care aides (N=35) from 2 government-owned and 2 private long-term care facilities in urban China. Directed and conventional content analysis were used, drawing upon core constructs of person-centered dementia care and Nolan’s (2006) senses framework. We found that although care aides were not trained in person-centered care, they did incorporate person-centeredness in their work by tailoring their care to the needs of older residents and facilitating interactions with residents and their peers through communication cues.

